# Invasive *Haemophilus influenzae* Type b (Hib) Infection in a Fully Vaccinated Child: A Case Report

**DOI:** 10.3390/diseases14060204

**Published:** 2026-06-07

**Authors:** Ho Yeung Lam, Shalomie Shadrach, Ben Brimblecombe, Hannah Woodall, Brianna Moss, Teresa McGorm, Rikki Graham, Mohana Rajmokan, Gino Micalizzi, Stephen B. Lambert

**Affiliations:** 1 Darling Downs Public Health Unit, Darling Downs Hospital and Health Service, Queensland Health, Toowoomba, QLD 4350, Australia; 2 Public Health Microbiology & Queensland Public Health and Infectious Diseases Reference Genomics, Public and Environmental Health Reference Laboratories, Pathology Queensland, Queensland Health, Brisbane, QLD 4108, Australia; 3 Public Health Intelligence Branch, Population Health Division, Queensland Health, Brisbane, QLD 4006, Australia; 4 Public Health Microbiology Laboratory, Public and Environmental Health Reference Laboratories, Pathology Queensland, Queensland Health, Brisbane, QLD 4108, Australia; 5 Communicable Diseases Branch, Population Health Division, Queensland Health, Brisbane, QLD 4006, Australia

**Keywords:** *Haemophilus influenzae* type b, epiglottitis, vaccine breakthrough infection, vaccine preventable diseases, whole-genome sequencing, case report

## Abstract

Background: Invasive *Haemophilus influenzae* type b (Hib) infection in children has become rare following the introduction of highly effective conjugate vaccines under national immunisation programmes. However, breakthrough invasive infections in fully immunised individuals can still occur. We report a case of invasive Hib infection presenting as epiglottitis and bacteraemia in a fully vaccinated 5-year-old boy in regional Queensland, Australia. Case presentation: The child, with no history of immunodeficiency, presented with a 3-day history of fever, progressive throat pain and reduced oral intake. Subsequent investigations revealed leukocytosis with left shift, markedly elevated C-reactive protein, and radiographic features consistent with epiglottitis. Blood culture grew *H. influenzae* type b. He was treated with intravenous cefotaxime and made a full recovery without complications. Immunological evaluation demonstrated Hib-specific IgG levels consistent with prior vaccinations, with normal immunoglobulin and lymphocyte profiles supporting the absence of immunodeficiency. Whole-genome sequencing of the isolate identified sequence type 6, a known circulating strain, and duplication of the capsule (*cap*-b) locus which has been associated with increased capsular polysaccharide production and reduced susceptibility to immune-mediated clearance. Conclusions: This case demonstrates that invasive Hib disease can occur in fully vaccinated, immunocompetent individuals and highlights the need for continued clinical vigilance. Pathogen-related factors, such as *cap*-b locus duplication, may reduce the effectiveness of the immune response. Despite this, immunisation can still confer partial protection, potentially contributing to the relatively mild clinical course. Ongoing surveillance and detailed microbiological investigation are essential to better understand and monitor vaccine breakthrough infections.

## 1. Introduction

Invasive disease caused by *Haemophilus influenzae* type b (Hib), including meningitis, epiglottitis, bacteraemia/septicaemia, and pneumonia, was historically a leading cause of morbidity and mortality among children. The introduction of conjugate Hib vaccines into childhood immunisation programmes has led to a substantial and sustained decline in disease incidence worldwide, including Australia [1]. Before introduction of the Hib vaccine into the Australian National Immunisation Program (NIP) in 1993, 549 Hib notifications were reported in Australia in 1992 alone, while only 345 notifications were reported between 2000 and 2017 [2].

Under the current Australian NIP schedule, the Hib-containing vaccine is administered as a primary series at 2, 4, and 6 months of age with either Infanrix Hexa (DTPa-HepB-IPV-Hib(PRP-T)) or Vaxelis (DT5aP-hepB-IPV-Hib(PRP-OMP)), followed by a booster dose at 18 months with a monovalent Hib (PRP-T) vaccine (ActHIB) [3]. The first dose may be administered from as early as 6 weeks of age and adjustment of the Hib vaccination schedule is not required for preterm infants [4]. Vaccine effectiveness is high after completion of the primary series, estimated at approximately 95% protection against invasive Hib diseases [5]. The booster dose induces a strong anamnestic immune response which contributes to sustained protection against invasive diseases [6,7].

In Queensland, Australia, Hib infection has been a notifiable condition since the early 1990s [8]. All laboratory confirmed cases are notified and then managed by the local public health unit (PHU) to enable timely epidemiological investigations and close contact management, as guided by the Communicable Diseases Network Australia (CDNA) national guidelines [9].

Despite the high effectiveness of vaccination, breakthrough infections in vaccinated individuals have been reported, although they remain rare. Invasive Hib disease can occur in fully vaccinated children, potentially due to several factors such as host immune response, vaccine failure, or pathogen-related factors [10]. Here we provide a case report of invasive Hib infection presenting as epiglottitis and bacteraemia in a fully vaccinated child in regional Queensland.

## 2. Case Description

### 2.1. Clinical Course

A 5-year-old boy, born at 36 weeks’ gestation and with a history of asthma, presented to a local emergency department (ED) with a 3-day history of cough, rhinorrhoea, and fever (Table 1). He was up to date with all vaccinations under the Australian NIP schedule, including Hib-containing vaccines. He received Infanrix Hexa at 6 weeks, 4 months, and 6 months of age, with an ActHIB booster administered at 19 months, in accordance with the NIP recommendations. On initial presentation, physical examination demonstrated a well-appearing child with mild throat erythema and fever. He was discharged with a diagnosis of upper respiratory tract infection and provided with analgesia.

He re-presented twice in the following 24 h with worsening malaise and reduced oral intake due to increasing throat pain, as well as noisy breathing reported by his mother. Although stridor, toxic appearance and tripod positioning were not present, reassessment showed progression of throat inflammation, clinical dehydration, and tender anterior cervical lymphadenopathy.

**Figure 1:**
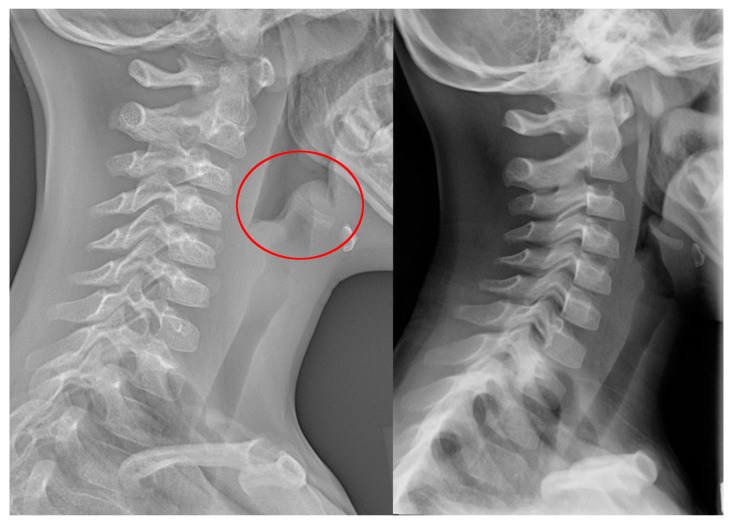
Lateral neck soft tissue radiographs demonstrating epiglottitis and its resolution. (**Left**): Radiograph on day 0 of admission showing epiglottic thickening with mild pharyngeal soft tissue distension (red ellipse). (**Right**): Radiograph on day 3 of admission showing resolution of swelling.

At the third presentation, blood tests demonstrated leukocytosis (16.9 × 10^9^/L; reference range: 5–15 × 10^9^/L) with neutrophilia (11.63 × 10^9^/L; reference range: 1.5–8 × 10^9^/L), appearance of metamyelocytes (2.62 × 10^9^/L; reference range: <0.01 × 10^9^/L) and a markedly elevated C-reactive protein (CRP, 195 mg/L; reference range: <5 mg/L). Chest radiography was unremarkable. X-ray of neck soft tissue showed epiglottic thickening with mild pharyngeal distension, suggestive of epiglottitis (Figure 1). He received a dose of dexamethasone and was commenced on intravenous cefotaxime, was admitted and subsequently transferred to a secondary hospital for further management.

Blood cultures collected at the third presentation grew *H. influenzae*. Organism identification was performed using an automated mass spectrometry microbial identification system (VITEK MS). β-lactamase production was assessed using a disc test (Cefinase), which was negative. Antimicrobial susceptibility testing was performed using E-test, with minimum inhibitory concentrations (MICs) determined for amoxicillin and cefotaxime. The isolate was susceptible to both amoxicillin (MIC 0.25 mg/L) and cefotaxime (MIC < 0.016 mg/L). Subsequently, the isolate was confirmed as type b using the slide agglutination serotyping method against the six capsular serotypes (a–f) and polymerase chain reaction targeting the capsular gene region specific to capsule type b [11]. There was no evidence of concurrent meningitis or pneumonia clinically or radiologically. A definitive diagnosis of Hib epiglottitis was made.

Following treatment, the patient showed clinical improvement with resolution of symptoms and tolerance of oral intake. By day 2 of admission white blood cell and CRP had begun to decline. Repeat blood culture on day 3 of admission was negative. He was discharged on day 10 of admission without complications.

A series of immunological investigations was performed on the day of discharge. Hib-specific IgG (anti-*Haemophilus influenzae* type b polyribosylribitol phosphate IgG) testing, measured using the VaccZyme™ Human Anti-Haemophilus Influenzae Type b Enzyme Immunoassay Kit (Binding Site Group Ltd., Birmingham, UK), demonstrated a protective antibody level of 1.2 μg/mL (>1.0 μg/mL equates with long-term protection [12]). Total IgG, IgM, and IgA levels were within normal limits. Lymphocyte counts and subpopulation profiles were also normal (both absolute and percentage values). There was no evidence of hyposplenism, supported by the absence of Howell–Jolly bodies on peripheral blood film and normal splenic appearance on abdominal ultrasonography.

### 2.2. Public Health Management

The local PHU was notified following identification of *H. influenzae* in blood culture. Contact tracing was initiated after confirmation of type b infection, with the exposure period defined as the seven days prior to symptom onset.

Three household contacts (all adults) were identified. All remained asymptomatic and none met criteria for vulnerable contacts (e.g., immunocompromised or asplenic individuals, infants aged younger than 7 months, or inadequately vaccinated children aged 7 months to 5 years). Accordingly, antibiotic chemoprophylaxis was not indicated.

The child did not attend childcare, and contact tracing in the school setting was not undertaken in accordance with CDNA guidelines, which state that secondary transmission of Hib in schools is uncommon in the post-vaccination era and primarily recommend contact management in household and childcare settings. All identified contacts were provided with public health advice and instructed to monitor for symptoms. Additional contact tracing conducted by the hospital infection control team did not identify any vulnerable patient or hospital staff contacts. No secondary cases were detected during the follow-up period.

### 2.3. Genomic Analysis

Whole-genome analysis of the *H. influenzae* isolate from blood culture was performed to assess potential reasons for vaccine failure. DNA was extracted from the isolate using the QiaSymphony DSP DNA Mini kit (Qiagen, Hilden, Germany) according to the manufacturer’s instructions. DNA concentrations were measured using Quant-iT™ High-Sensitivity dsDNA Assay Kit as per the manufacturer’s instructions (Thermo Fisher Scientific, Waltham, MA, USA), normalised to 0.3 ng/μL, and stored at −20 °C prior to library preparation. Genomic libraries were prepared with the Nextera XT DNA sample preparation kit (Illumina, San Diego, CA, USA) and sequenced on the NextSeq500 with the 500 Mid Output v2.5 kit (300 cycles) (Illumina, San Diego, CA, USA) according to the manufacturer’s instructions.

A total of 9,184,908 sequence reads were generated with an average sequence length of 138 bp. This gave 683× coverage of the *H. influenzae* 10810 reference genome (Genbank accession number FQ312006). Genomic sequences were trimmed using trimmomatic (v0.36) using default parameters and de novo assembled into contigs using the SPAdes assembler (v3.13.1) with default parameters. A total of 49 contigs were generated by the assembly with an N50 of 127,348 bp and total assembly size of 1,879,989 bp. Sequences are available at Genbank under accession number PRJEB113677. Multilocus sequence typing (MLST) analysis was then performed on the contigs using the *H. influenzae* MLST scheme (current as of 14 June 2025) developed by PubMLST [13] with Ridom SeqSphere+ (v10.5.2). MLST analysis demonstrated that the isolate belonged to sequence type 6 (ST6).

Whole-genome sequencing confirmed the identification of Hib. The locus responsible for capsule production (*cap*-b), was investigated by manual inspection of the assembly in Geneious R11 and comparison with the *H. influenzae* 10810 strain as a reference. This analysis revealed duplication of the entire capsule locus in a single contig, with two copies of each gene present. The *cap*-b locus was further assessed for the presence of single-nucleotide polymorphisms (SNPs) in genes involved in capsular polysaccharide biosynthesis and export when compared to the *H. influenzae* 10810 strain [14]. No SNPs were detected in these regions from this isolate, which were identical to those for the *H. influenzae* 10810 strain.

**Figure 2:**
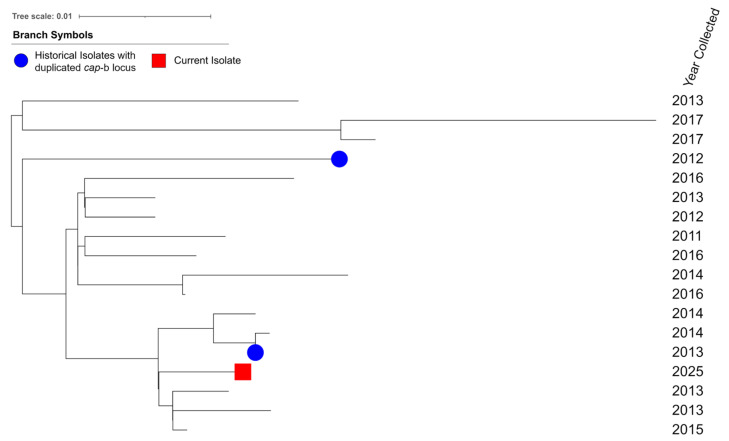
Neighbour-joining phylogenetic tree built using differences in 941 genes. The isolate described in this case is indicated on the tree with a red rectangle. Other isolates that also had a duplicated *cap*-b locus are indicated with blue circles. All other isolates included on the tree had a single *cap*-b locus; year of collection is indicated to the right of the tree. Branch length represents genetic distance as indicated by the scale bar.

The *cap*-b loci of 17 historical invasive ST6 *H. influenzae* isolates from the Public and Environmental Health Reference Laboratories (Pathology Queensland) culture collection, collected in Queensland between 2011 and 2017, were investigated. Two isolates, collected in 2012 and 2013, were found to also have a duplicated *cap*-b locus. Phylogenetic analysis of all of these ST6 isolates using core genome MLST found that none of them, including the two with *cap*-b locus duplication, had a high level of genetic similarity (as defined by <5 allele differences) to the isolate from the current case (Figure 2). Overall, the isolate from the current case had between 8 and 62 allele differences to the other isolates included in the analysis, whereas the allelic differences among all 17 historical isolates ranged from 1 to 71.

## 3. Discussion

In the post-vaccine era, invasive Hib infection in children is rare and predominantly reported in those who are incompletely vaccinated or have underlying medical conditions, such as prematurity, immunodeficiency, or malignancy [15,16]. The present case is notable because it occurred in a child who had documented receipt of a complete Hib vaccination schedule in accordance with the Australian NIP recommendations with no evidence suggestive of immunodeficiency, thereby providing an opportunity to explore possible mechanisms of vaccine breakthrough infection.

Vaccine-related factors, such as reduced vaccine potency from cold chain breaches, are unlikely in this case given the robustness of the immunisation management system in Australia and the presence of Hib-specific IgG, which suggested that the vaccines appeared to have elicited an appropriate serological response.

However, while vaccine-related failure and overt immunodeficiency are unlikely, these findings do not fully exclude subtle or functional immunological factors that may have increased the patient’s susceptibility to invasive Hib disease. Although no evidence of immunodeficiency was identified in this patient (protective Hib-specific IgG level, normal total immunoglobulin levels and lymphocyte subsets and no evidence of hyposplenism), a limitation should be acknowledged in that some additional investigations recommended following invasive encapsulated bacterial infections were not performed. These included human immunodeficiency virus serology, complement testing (CH50, AH50, C3, and C4), and mannose-binding lectin levels [17].

Furthermore, the normal total immunoglobulin levels and lymphocyte subsets do not fully capture functional humoral and cellular immunity. The possibility of transient functional antibody impairments cannot be excluded, although they typically occur in children less than 2 years of age [18] or present with recurrent respiratory infections [19]. On the other hand, the demonstration of a protective level of Hib-specific IgG also has limitations, as effective immunity against Hib also depends on functional antibody characteristics, including avidity [20], which was not measured in this case. The Hib-specific IgG level in this case was measured on day 14 after symptom onset and may therefore reflect an anamnestic response rather than baseline pre-infection immunity.

In the absence of evidence suggestive of immunodeficiency, pathogen-related factors may therefore have contributed to breakthrough infection in this case. Currently, ST6 is one of the most frequently reported clonal complexes identified in Hib isolates causing invasive diseases across multiple age groups and geographical regions. These include Italy [21], the Republic of Ireland [22], Denmark [23], Argentina [24], the United Kingdom [25], Canada [26], Japan [27], the United States [28], and France [29], as well as Queensland, Australia [30]. The isolate in the present case belonged to ST6, indicating the infection was caused by a known globally and locally circulating clonal complex associated with invasive disease, rather than a novel or atypical one. Nevertheless, phylogenetic analysis demonstrated that the current isolate was not closely related to historical Queensland ST6 isolates.

Notably, whole-genome analysis has identified duplication of the *cap*-b locus. Hib vaccines confer protection through antibodies directed against the capsular polysaccharide which is a key virulence factor [31]. Previous studies have shown that duplication of the *cap*-b locus can result in increased capsule production, leading to higher capsule density of polysaccharides and reduced susceptibility to complement-mediated lysis [32,33]. Hib strains with multiple copies of the *cap*-b locus have also been associated with more severe invasive disease [34]. It has been hypothesised that such strains may require higher antibody concentrations for effective immune system containment [35]. Nevertheless, a recent study in France reported that 37.0% of Hib isolates (71/191) from children under 5 years of age carried capsular duplication, and found no evidence of an association with vaccine failure. However, the authors noted that interpretation was limited by the lack of sufficient pre-vaccine era isolates for comparison [29]. Data on the prevalence and clinical significance of *cap*-b locus duplication among invasive Hib isolates in Australia remain limited. Further research is therefore needed to clarify its distribution and potential association with virulence and breakthrough invasive infections.

In this case, the presence of a protective Hib-specific IgG level likely indicates that vaccine-induced immunity was achieved; however, the increased capsule expression from the *cap-b* locus duplication may have reduced the effectiveness of this response. Nevertheless, the relatively mild clinical course suggests that vaccination may have conferred partial protection despite the occurrence of invasive disease. Taken together, these findings support a potential role for pathogen-related factors, particularly the *cap-b* locus duplication, in causing the breakthrough infection in this patient, while acknowledging that subtle host immune factors not captured by routine testing could not be completely excluded. Epidemiological and laboratory investigations were unable to identify the possible source of infection.

## 4. Conclusions

Overall, this case underscores the importance of continued epidemiological and molecular surveillance of Hib disease in the post-vaccine era [36]. Attention should be paid to genomic features associated with increased virulence and breakthrough infection, including *cap*-b locus duplication. Ongoing monitoring of the frequency, distribution, and clinical association of these features may provide early signals of changes in disease epidemiology. In cases of suspected vaccine failure, comprehensive assessment for underlying immunodeficiency and detailed microbiological and molecular investigation, including whole-genome sequencing, are warranted to better elucidate the underlying cause [10,16]. Clinically, this case highlights that invasive Hib infection, including epiglottitis, can still occur in fully vaccinated children. Early presentations may be non-specific. Clinicians should maintain a high index of suspicion, with appropriate reassessment and safety-netting to ensure timely recognition and management of potentially rapidly progressive disease.

## Figures and Tables

**Table 1:** Clinical timeline showing symptom onset and hospital admission, with serial key microbiological, laboratory, and radiological findings. Abnormal results are shown in italics.

Day of Symptom Onset	Day 0	Day 3	Day 4	Day 6	Day 7	Day 14
Day of Hospital Admission	-	-	Day 0	Day 2	Day 3	Day 10
Key events	Symptom onset	First and second ED presentations	Third attendance at ED; transferred and admitted to regional hospital	-	-	Discharged home
Microbiological investigation	-	-	*Blood culture* *(later positive for Hib)*	-	Blood culture (negative)	-
White cell count (reference range: 5–15 × 10^9^/L)	-	-	*16.9 × 10^9^/L*	10.8 × 10^9^/L	14.3 × 10^9^/L	9.1 × 10^9^/L
Neutrophils (reference range: 1.5–8 × 10^9^/L)	-	-	*11.63 × 10^9^/L*	*8.98 × 10^9^/L*	7.33 × 10^9^/L	4.66 × 10^9^/L
Metamyelocytes (reference range: <0.01 × 10^9^/L)	-	-	*2.62 × 10^9^/L*	Absent	Absent	Absent
C-reactive protein (reference range: <5 mg/L)	-	-	*1* *95 mg/L*	*63 mg/L*	-	1.2 mg/L
Chest radiograph	-	-	Unremarkable	-	-	-
X-ray of neck soft tissue	-	-	*Epiglottic thickening with mild pharyngeal distension, suggestive of epiglottitis*	-	Resolution of swelling	-

## Data Availability

Data will be made available upon request.

## Abbreviations

The following abbreviations are used in this manuscript:

Hib	*Haemophilus influenzae* type b
NIP	National Immunisation Program
PHU	Public health unit
CDNA	Communicable Diseases Network Australia
ED	Emergency department
CRP	C-reactive protein
MLST	Multilocus sequence typing
SNPs	Single-nucleotide polymorphisms
